# Spatial Nano-Morphology of the Prolamellar Body in Etiolated *Arabidopsis thaliana* Plants With Disturbed Pigment and Polyprenol Composition

**DOI:** 10.3389/fcell.2020.586628

**Published:** 2020-10-08

**Authors:** Michał Bykowski, Radosław Mazur, Daniel Buszewicz, Joanna Szach, Agnieszka Mostowska, Łucja Kowalewska

**Affiliations:** ^1^Department of Plant Anatomy and Cytology, Institute of Plant Experimental Biology and Biotechnology, Faculty of Biology, University of Warsaw, Warsaw, Poland; ^2^Department of Metabolic Regulation, Institute of Biochemistry, Faculty of Biology, University of Warsaw, Warsaw, Poland; ^3^Institute of Biochemistry and Biophysics, Polish Academy of Sciences, Warsaw, Poland

**Keywords:** etioplast, prolamellar body, protochlorophyllide, cubic membranes, electron tomography, carotenoids, polyprenols

## Abstract

The prolamellar body (PLB) is a periodic bicontinuous membrane structure based on tubular tetrahedral units. PLBs are present in plant etioplasts and, upon illumination, directly transform into the lamellar thylakoid networks within chloroplasts. Efficient tubular-lamellar rearrangement and later formation of the photosynthetically active thylakoid membranes are crucial steps in the development of plant autotrophy. PLB membranes are mainly composed of galactolipids, carotenoids, and protochlorophyllide (Pchlide), the chlorophyll precursor, bound in a complex with NADPH and Pchlide oxidoreductase. Although the PLB structure has been studied for over 50 years, the direct role of particular membrane components in the formation of the PLB paracrystalline network remains elusive. Moreover, despite the numerous literature data regarding the PLB geometry, their reliable comparative analysis is complicated due to variable experimental conditions. Therefore, we performed comprehensive ultrastructural and low-temperature fluorescence analysis of wild type *Arabidopsis thaliana* (Arabidopsis) seedlings grown in different conditions typical for studies on etiolated seedlings. We established that the addition of sucrose to the growing media significantly affected the size and compactness of the PLB. The etiolation period was also an important factor influencing the PLB structural parameters and the ratio of free to complex-bound Pchlide. Thus, a reliable PLB structural and spectral analysis requires particular attention to the applied experimental conditions. We investigated the influence of the pigment and polyprenol components of the etioplast membranes on the formation of the PLB spatial structure. The PLB 3D structure in several Arabidopsis mutants (*ccr1-1*, *lut5-1*, *szl1-1npq1-2*, *aba1-6, pif1*, *cpt7*) with disturbed levels of particular pigments and polyprenols using electron tomography technique was studied. We found that the PLB nano-morphology was mainly affected in the *pif1* and *aba1-6* mutants. An increased level of Pchlide (*pif1*) resulted in the substantial shift of the structural balance between outer and inner PLB water channels and overall PLB compactness compared to wild type plants. The decrease in the relative content of β-branch xanthophylls in *aba1-6* plants was manifested by local disturbances in the paracrystalline structure of the PLB network. Therefore, proper levels of particular etioplast pigments are essential for the formation of stable and regular PLB structure.

## Introduction

The prolamellar body (PLB) is a unique periodic bicontinuous membrane structure of angiosperm etioplasts. It is formed mainly in young, light-deprived tissues having a photosynthetic potential when exposed to light (reviewed in Solymosi and Aronsson, 2013; Pogson et al., 2015; Kowalewska et al., 2019). Therefore, PLBs are persistent structures of plastids in cotyledons or the first true leaves of dark-germinating seedlings, but are also present in, e.g., close buds of some species (Solymosi and Böddi, 2006; Solymosi and Schoefs, 2010). Although the PLB was visualized for the first time already in the 1950s (Leyon, 1954), but still the knowledge about the structural pathway of its formation remains elusive and requires further investigation. On the other hand, the on-light transformation of the paracrystalline PLB into the lamellar structure of grana and stroma thylakoids is well known (Gunning, 1965; Forger and Bogorad, 1973; Bradbeer et al., 1974; Robertson and Laetsch, 1974; Mostowska, 1986; Rudowska et al., 2012), however, the spatial structural details of the tubular-lamellar membrane transformation during the chloroplast biogenesis was only recently shown (Kowalewska et al., 2016). Thus, the PLB is a direct precursor of one of the most complicated and important membrane systems in nature, i.e., the thylakoid network of chloroplasts hosting light-dependent reactions of the photosynthesis. Due to a specific composition and the structural configuration of an exceptionally high surface-to-volume ratio (Gunning, 2001), the PLB is considered to play various functions during the chloroplast development. For instance, PLB is a lipid reservoir for developing thylakoids, and a significant increase in *de novo* lipid synthesis was detected only when no remnants of PLB were visible (Armarego-Marriott et al., 2019). Moreover, we have shown previously that the presence of large and stable PLB correlates with a highly efficient formation of grana structures during the early stages of the chloroplast biogenesis (Kowalewska and Mostowska, 2016).

More generally, PLB, similar to cubic membranes in other configurations, separate aqueous phase into a two-channel system enabling their different molecular composition and, therefore, function. The size of the water channels, controlled by the scale of the structure, can exclude or enable the localization of certain molecules on the particular side of the membrane (Mezzenga et al., 2019). In such way, we can directly link the ultrastructural features of the PLB, length-scale in particular, with new possible biological functions of this membrane arrangement performed on the molecular level. At the 3D level the balance between channels can be expressed as a ratio of inner and outer volumes of the PLB lattice modeled region.

The PLB structure is in general based on the triply periodic minimal surface template; however, it is characterized by the asymmetry leading to a geometrical imbalance between two sides of the membrane (Mezzenga et al., 2019). The majority of PLB configurations is formed via repetition of a basic tetrahedral tubular element forming 3D hexagonal lattice with the same symmetry as the zinc sulfide crystal – wurtzite or zincblende (Gunning and Steer, 1975; Murakami et al., 1985; Lindblom and Rilfors, 1989; Lindstedt and Liljenberg, 1990; Williams et al., 1998; Selstam et al., 2007). PLB can also adapt a geometry based on different polyhedrons forming so-called “open” type arrangements (Gunning, 2001). It is worth mentioning that even PLBs having the same geometrical configuration can markedly differ in terms of length-scale and balance between different structural features like the tubule diameter or hexagon size.

The paracrystalline PLB structure is determined by its specific composition; however, the role of only several components of the internal etioplast network in the formation and maintenance of such unique cubic arrangement has been identified so far. The crucial role of polar lipids – monogalactosyldiacylglycerol (MGDG) and digalactosyldiacylglycerol (DGDG) in this process was recently shown by Fujii et al. (2017, 2018, 2019) using *dgd1* mutant and DEX-inducible amiR-*MGD1* line. It was proposed already in earlier studies, that the proper MGDG/DGDG ratio could play a role in the PLB organization (Selstam and Sandelius, 1984). Such hypotheses were mainly based on different roles of the conically shaped MGDG and the bilayer-forming DGDG in the bending of etioplast membranes (Demé et al., 2014). Etioplasts with a decreased MGDG and DGDG levels exhibit a severe decrease in the PLB size and show aberrations in its geometrical configuration (Fujii et al., 2017, 2018).

Apart from lipid components, the PLB also contains over 60 proteins from, e.g., pigment biosynthesis pathways, Calvin–Benson–Bassham components, and thylakoid photosynthesis proteins (Dehesh and Ryberg, 1985; Blomqvist et al., 2008). The most abundant protein is the light-dependent protochlorophyllide oxidoreductase (LPOR) enzyme; its key role in the formation of PLB has been previously shown using the *porA-1* mutant (Paddock et al., 2012). The accumulation of LPOR proteins can restore PLBs in the constitutive photomorphogenic (*cop1*) mutants highlighting the central role of LPOR in the maintenance of the PLB structure (Sperling et al., 1998). The LPOR protein forms a ternary complex with NADPH and protochlorophyllide (Pchlide) – chlorophyll precursor, the most abundant pigment of PLB membranes (Armstrong et al., 1995; Blomqvist et al., 2008). Plants defective in the Pchlide accumulation do not develop PLBs (Von Wettstein et al., 1995; Franck et al., 2000), which might be directly related to lack of the Pchlide:LPOR:NADPH complex associated previously with the PLB structure formation (Böddi et al., 1989; Lebedev et al., 1995; Sperling et al., 1997; Selstam et al., 2011; Myśliwa-Kurdziel et al., 2013). It was shown that the LPOR abundance is proportional to the photoactive (bound in the ternary complex) Pchlide content, which correlates with the increased ratio of the photoactive to non-photoactive (free) Pchlide and with the PLB size (Franck et al., 2000). High photoactive/non-photoactive Pchlide ratio was also registered for seedlings germinated on a medium with sucrose (Suc) (e.g., Samol et al., 2011; Fujii et al., 2017) compared to these etiolated on a medium solution without additional carbon source (Franck et al., 2000; Huq et al., 2004; Aronsson et al., 2008). However, no correlation of such results with PLB structural parameters was given. Reliable analysis of the literature data linking the ultrastructural and spectral level of the PLB organization is particularly difficult due to various experimental setups used and lack of systematic analysis that could possibly show the influence of the nutrition media and the etiolation time on the PLB formation.

Carotenoids form another group of molecules that are highly abundant in the PLB membrane fraction. Their structural role in the PLB formation was demonstrated in the PLB-deficient *ccr2* mutant, which accumulates particular poly*-cis* carotenoids and maintains wild type levels of LPOR and Pchlide (Park et al., 2002; Cuttriss et al., 2007; Cazzonelli et al., 2020). In the *ccr2* mutant, restoration of the PLB structure is possible due to further mutations in the ζ-carotene isomerase (ZISO) pointing directly to the role of the *cis*-carotenoid component in the PLB structure development (Cazzonelli et al., 2020). However, because carotenoids are a large and diverse group of the lipophilic pigments, the role of other molecules from this group in the PLB formation cannot be excluded and remains to be studied. Finally, PLB and developed thylakoid membranes share similarities in composition, especially in terms of lipids (Selstam and Sandelius, 1984). Nonetheless, the role of many membrane components in the formation of grana and stroma thylakoid structure has been proposed, while their role in the PLB structure development still remains elusive, e.g., curvature-inducing proteins from CURT1 family or plastidial polyprenols (linear polyisoprenoids structurally similar to carotenes) (Armbruster et al., 2013; Akhtar et al., 2017).

The main goal of this study was to reveal the structural role of different pigments and polyprenols localized in the PLB membranes in the formation of their ordered bicontinuous configuration. Moreover, we checked the influence of experimental setup on the PLB formation and spectral properties of *Arabidopsis thaliana* (Arabidopsis) etioplasts. We established that changes in the experimental conditions substantially influence the etioplast development. Such observation might explain ambiguity in the previously published results regarding the PLB structure and its correlation with the Pchlide spectra pattern in the wild type and different Arabidopsis mutants. The 2D and 3D analyses of the PLB arrangement in pigment and polyprenol deficient Arabidopsis mutants pointed to a significant influence of Pchlide and β-β-xanthophylls on the PLB nano-morphology. Finally, we gave a direct experimental evidence that spatial parameters of PLB might be reliably predicted from the generated 3D theoretical models based only on measurements of the 2D TEM cross-sections.

## Materials and Methods

### Growth Conditions

Seeds of *Arabidopsis thaliana* mutants *ccr1-1* (N68151, *sdg8*; Park et al., 2002), *lut5-1* (N616660, SALK_116660; Kim and DellaPenna, 2006), *szl1-1npq1-2* (N66023; Li et al., 2009), *aba1-6* (N3772; Niyogi et al., 1998), *pif1* (N66041; Huq et al., 2004), *cpt7* (N48213, SALK_022111; Akhtar et al., 2017), and ecotype Col-0 (N1092, wild type) were obtained from The European Arabidopsis Stock Center. Seedlings were grown in Petri dishes on Murashige and Skoog Basal Medium and Gamborg’s vitamins supplemented with 0.8% Phytagel^TM^ (P8169, Sigma-Aldrich). For particular experiments, the nutrition medium was additionally supplemented with 1% of Suc. Etiolation (3–6 days in 23°C) was preceded with a 24 h stratification in 4°C and 4 h illumination (120 μmol photons m^–2^ s^–1^ in 23°C) to induce germination. All samples were collected in the darkness with photomorphogenetically inactive dim green light.

### Transmission Electron Microscopy (TEM)

Samples for TEM were fixed for 2 h in 2.5% glutaraldehyde in 50 mM cacodylate buffer (pH 7.4), postfixed in 2% OsO_4_ at 4°C overnight and dehydrated in a graded series of acetone using the following sequence: 10 min, 30% (v/v); 10 min, 40% (v/v); 10 min, 60% (v/v); 30 min, 70% (v/v); 40 min, 80% (v/v); 3 × 40 min, 100%. Dehydrated samples were infiltrated in acetone:resin mixtures (3:1; 1:1; 1:3) and finally embedded in a pure resin medium (Agar 100 Resin Kit). Blocks with samples were cut into 90 nm specimens using the Leica UCT ultramicrotome. TEM images were performed with the help of the JEM 1400 (JEOL) equipped with Morada G2 CCD camera (EMSIS GmbH) in the Laboratory of Electron Microscopy, Nencki Institute of Experimental Biology of Polish Academy of Sciences, Warsaw, Poland. The PLB ultrastructural characteristics were calculated with the help of ImageJ software (Abramoff et al., 2004). PLB cross-sectional area was measured from 2D images of whole etioplasts using manual polygon tracing of PLB cubic region visible on the micrograph (Supplementary Figure 1A). Periodicity parameter was calculated based on averaged values obtained from Fast Fourier Transform (FFT) of 2D cross sections of micrographs showing nearly hexagonal planar lattice (Supplementary Figures 1B–D). PLB tubule width was established via manual measurement of the diameter of PLB tubules visible in particular orientations of PLB cross sections (examples show in Supplementary Figure 1E); measurements were based on the outer limits of each tubule (Supplementary Figure 1F).

### Electron Tomography (ET) and 3D Theoretical Models

For ET experiments, samples were fixed as described above, and 200 nm thick leaf sections were placed on 100 mesh nickel grids for further analysis. Tomograms were collected from +60° to −60° at the 1° increment around one axis using the same JEM 1400 electron microscope equipped with the tomography supply at the voltage of 120 kV. Aligned tomogram tilts were reconstructed by 100 iterations of the SIRT algorithm in the TomoJ (ImageJ plugin) software (Messaoudii et al., 2007). PLB segmentations were done via generation of PLB membrane isosurfaces in the Imaris 8.4.2 software (Bitplane AG) (surface detail 3.5 nm, background subtraction 6.2 nm). Spatial structural parameters were calculated using the MeasurementPro package of Imaris 8.4.2. Area and inner volume of PLB isosurface were automatically generated from the obtained model, while outer volume was calculated as a volume of the space outside the isosurface placed inside the cuboid of the modeled region (Supplementary Figures 1G–I). Theoretical 3D models were generated using the Autodesk Fusion 360 software based on the exact structural parameters obtained from TEM cross-sections.

### Low-Temperature Fluorescence (77 K)

Steady-state low-temperature (77 K) fluorescence emission spectra of Pchlide were recorded using a modified Shimadzu RF-5301PC spectrofluorometer with optical fibers guiding the excitation and emission beams (fluorescence emission detection at 0° angle). Freshly collected seedling samples (*n* ≈ 50–100 plants per single measurement) were tightly packed and placed directly between polytetrafluoroethylene and non-fluorescent glass plates of 15 mm diameter. Sample holder was submerged in liquid nitrogen and scans were taken in the range of 600–800 nm and 1 nm interval through the LP600 filter. The excitation wavelength was set at 440 nm, the excitation and emission slits were set at 10 and 5 nm, respectively. Raw spectra were subtracted using spectrum obtained for empty sample holder (both plates), smoothed and normalized to the value indicated in the particular figure captions.

### Pigment Extraction and Chromatography

Pigments were extracted from cotyledons and upper parts (∼2–4 mm) of seedling hypocotyls (n ≈ 200) in 1 mL acetone:ethyl acetate 3:2 (v/v) as described in Cuttriss et al., 2007. Extracted pigments were separated using the Shimadzu Prominence HPLC System with PDA detector on Atlantis C-18 (4.6 × 250 mm, 5 μm, 100 Å) column (Waters). Elution was performed using ethyl acetate gradient in acetonitrile:water:triethylamine 9:1:0.01 (v/v) at 1 mL min^–1^ for 40 min according to the following timetable (0–2 min, 0% ethyl acetate; 2–32 min 0–66.7%, 32.2–37 min 66.7–100%). Compounds were identified, and the HPLC peak areas were integrated based on absorption spectra and retention times, according to Cuttriss et al. (2007); peak areas were integrated at 456 nm.

### Polyprenol Extraction and Chromatography

The procedure of polyprenol extraction and analysis was performed as described in Akhtar et al. (2017). Briefly, 350 mg of snap-frozen etiolated seedlings were grounded in liquid nitrogen using mortar and pestle, suspended in a mixture of chloroform:methanol 1:1 (v/v), supplemented with 10 μg of internal standard (Prenol-27; Collection of Polyprenols, Institute of Biochemistry and Biophysics, Polish Academy of Sciences) and incubated for 72 h at 4°C in darkness. The extracts were filtered, evaporated under a stream of nitrogen, and hydrolyzed for 1 h at 95°C in a mixture of toluene:7.5% KOH (in water):95% ethanol 20:17:3 (v/v). Next, lipids were extracted three times with hexane, applied to a silica gel 60 column, and purified using the isocratic elution with 20% (v/v) diethyl ether in hexane. Polyprenols were analyzed by HPLC, as described previously (Skorupińska-Tudek et al., 2003). Extracts were separated by HPLC (Waters) using a ZORBAX XDB-C18 (4.6 × 75 mm, 3.5 μm) reverse-phase column (Agilent). Polyprenols were eluted with a linear gradient from 0% to 100% of methanol:isopropanol:hexane 2:1:1 (v/v) in water:methanol 1:9 (v/v) at a flow rate of 1.5 mL/min. Polyprenols were detected by absorption at 210 nm and quantified relative to the internal standard.

### Statistical Analysis

For determination of the statistical significance of differences between results, one-way ANOVA with *post hoc* Tukey test at *p* = 0.05 was applied. All experiments were performed in three repetitions at least. Structural cross-sectional features were calculated for ≥ 20, while 3D spatial parameters from three modeled regions of interest per each variant.

## Results

In the first part of this study, we addressed the literature discrepancies regarding the correlation of PLB structure with etioplast spectral properties by testing the sampling methods, type of nutrition media, and time of etiolation. Further, after the selection of preferable experimental setup, we performed detailed 2D and 3D structural analysis of PLBs in plants with altered levels of polyprenols and different pigments, present in etiolated tissue.

### Etioplast Distribution in Arabidopsis Seedlings

**FIGURE 1:**
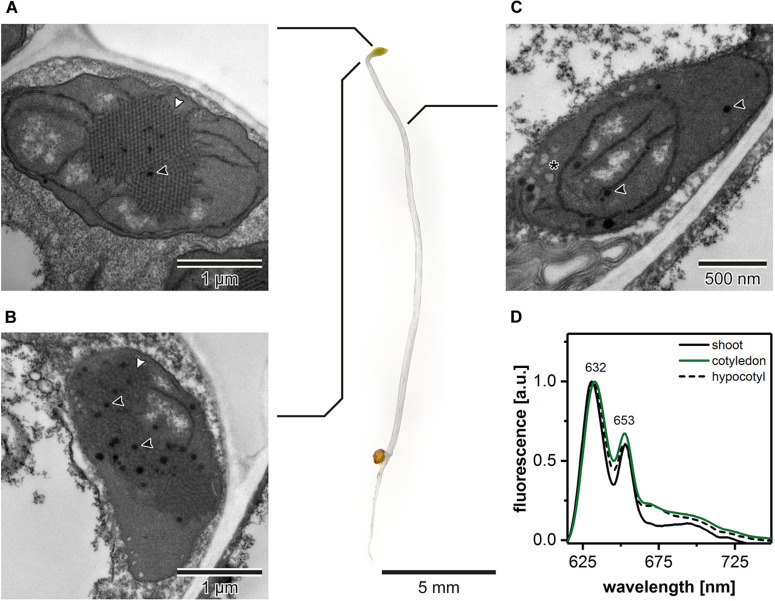
Ultrastructure of plastids in different shoot regions of 5-day etiolated Arabidopsis (Col-0) seedling grown on Murashige and Skoog medium without sucrose supplementation; white arrowhead – prolamellar body (PLB), black arrowhead – plastoglobule, asterisk – vesicle **(A–C)**. Representative low-temperature (77 K) fluorescence emission spectra (excitation at 440 nm) of shoot (hypocotyl and cotyledons), cotyledons and upper 2 mm of hypocotyl, and hypocotyl **(D)**; all spectra were normalized to the maximal value; note that the same, representative Col-0 “cotyledon” spectra (green) is also presented in Figures 3, 4, 6 – color code is preserved throughout the manuscript.

The ultrastructural analysis of plastids in subsequent regions of the shoot in 5-day etiolated Arabidopsis seedlings (Figure 1) revealed that etioplasts, characterized by the presence of paracrystalline PLB structure, were located in cotyledons and the first 2 mm of upper hypocotyl part (Figures 1A,B). However, etioplasts were more abundant in TEM cross-sections of cotyledons, and the regularity of the PLB located in this part of the seedling was higher compared with the upper hypocotyl part. In the middle and lower parts of the shoot, we did not observe PLB in the plastid cross-sections; only porous prothylakoids, vesicular structures, and plastoglobules were visible. This result points to the presence of proplastids in the middle and lower hypocotyl sections (Figure 1C). Moreover, we measured the 77 K emission spectra of Pchlide (i) in the total shoot fraction, (ii) in cotyledons and small hypocotyl parts (both including etioplasts), as well as (iii) in the middle and lower hypocotyl parts enriched with proplastids (Figure 1D). All analyzed spectra had two characteristic bands (∼632 and ∼653 nm). The first one corresponded to non-photoconvertible, free Pchlide species, while the second was related to the Pchlide:LPOR:NADPH complex (e.g., Ryberg and Sundqvist, 1982; Schoefs and Franck, 2003, 2008). Slight differences in the 632/653 nm peak ratios were observed between samples from different seedling regions. The higher signal coming from Pchlide:LPOR:NADPH complex was registered in cotyledon samples, which correlates with the PLB presence registered in TEM data (Figures 1A,B,D). For all further experiments, we decided to collect seedling regions enriched with etioplasts only. Moreover, all TEM and tomography data were obtained from the central part of the cotyledon blade.

### The Role of Nutrition Media in the Formation of the PLB Structure

**FIGURE 2:**
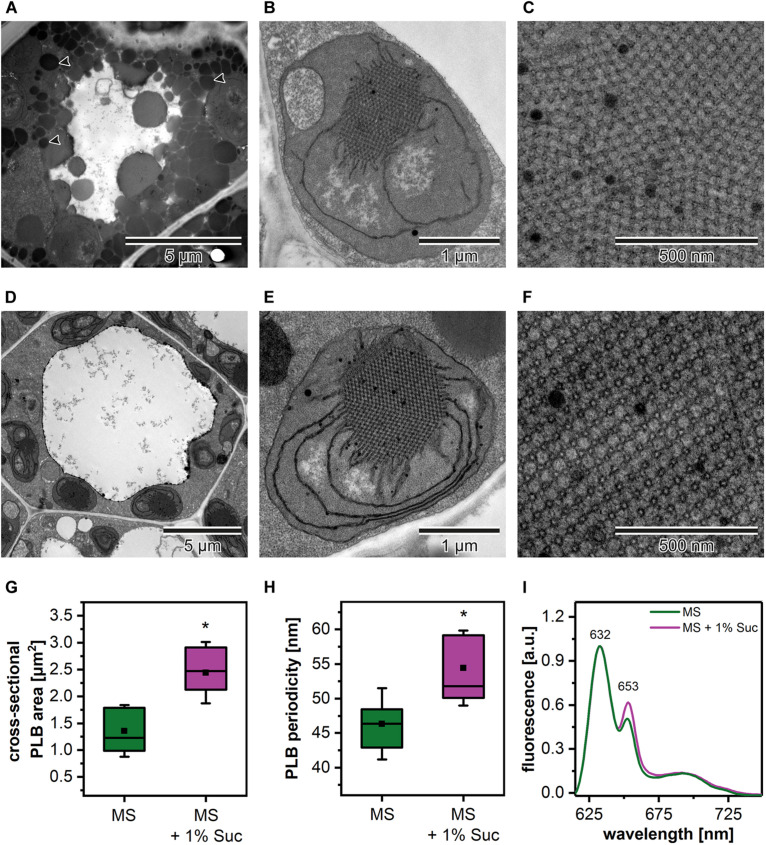
Cotyledon ultrastructure of 5-day etiolated Arabidopsis (Col-0) seedlings grown on Murashige and Skoog (MS) medium without **(A–C)** and with sucrose (1% Suc) supplementation **(D–F)**; electron micrographs showing whole cell (black arrowhead – lipid body) **(A,D)**, etioplast ultrastructure **(B,E)**, and prolamellar body (PLB) nano-morphology **(C,F)**. Measurements of the PLB structural parameters **(G,H)**; the horizontal line and black square in each box represent the median and mean value of the distribution, respectively; the bottom and top of each box represent 25 and 75 percentile; whiskers denotes standard deviation (SD); results from MS + 1% Suc samples marked with asterisk differ significantly at *p* = 0.05 from MS samples. Representative low-temperature (77 K) fluorescence emission spectra (excitation at 440 nm) of cotyledons and upper ∼ 2 mm of hypocotyl **(I)**; all spectra were normalized to the maximal value.

Due to the fact that the etiolation and de-etiolation process in Arabidopsis is studied using nutrition media supplemented with 1–2% Suc or depleted in an additional carbon source, we decided to test how such variability in growing conditions could influence the PLB structure and etioplast spectral properties (Figure 2). We established that the Suc supplementation resulted in an increased rate of the storage lipid degradation, i.e., no lipid bodies were visible in TEM sections of cotyledon cells (Figures 2A,D). Moreover, the addition of Suc was correlated with a markedly larger PLB size (Figures 2B,E,G and Supplementary Figure 1A) and an increased periodicity (Supplementary Figures 1B–D) of the PLB lattice compared with plants etiolated on a medium without sugar supplementation (Figures 2C,F,G). Periodicity is a parameter describing distances (nm) between the neighboring cross-sectional unit cells of the PLB hexagonal lattice; the higher the value, the more loose the PLB structure. Changes in the PLB structure were related with the fluorescence results in which larger PLB coincided with an increased contribution of the 653 nm band in the Pchlide emission spectra (Figures 2G,I). Although lack of lipid bodies in the cell cytoplasm is a factor that makes TEM analyses easier, we decided to use Suc-free nutrition media to follow a more natural pattern of seedling growth. Sugar metabolism is very complex and can influence different aspects of seedling development, including lipid and protein composition. Moreover, sugars also serve as signaling molecules, which can severely affect different metabolic pathways playing a central role in plastid biogenesis (Tognetti et al., 2013; Lastdrager et al., 2014).

### The Size of the PLB and Etioplast Spectral Properties During the Etiolation Process

**FIGURE 3:**
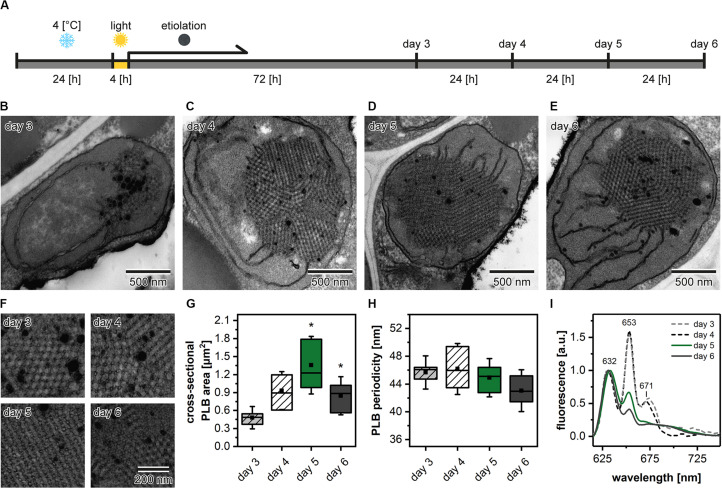
Scheme of the applied experimental setup. After stratification in 4°C plants were grown in the optimal (22°C) temperature **(A)**. Ultrastructure of cotyledon etioplasts **(B–E)** and prolamellar bodies (PLB) **(F)** of 3- to 6-day etiolated Arabidopsis (Col-0) seedlings; all PLBs **(F)** are shown in identical magnification indicated in the lower right panel. Measurements of the PLB structural parameters **(G,H)**; description of the chart box as in Figure 2; results marked with asterisk on the top of the boxplot differ significantly at *p* = 0.05 from the previous day of etiolation. Fluorescence emission spectra (excitation at 440 nm) of cotyledons and the upper 2 mm of hypocotyl **(I)**; all spectra were normalized to 1 at 632 nm.

Etiolation of Arabidopsis seedlings is a process that requires preceding stratification and exposition of seeds to light to induce the coordinated germination of seedlings (Figure 3A). We analyzed the PLB structure and its spatial properties in plants etiolated for 3, 4, 5, and 6 days to establish how does the etiolation time influence the etioplast development. We registered the PLB-containing etioplasts in cotyledons of all examined seedlings (Figures 3B–E). The area of PLB increased gradually up to 5 days of etiolation and decreased markedly during the last day of the experiment (Figure 3G). The PLB periodicity slightly decreased during the etiolation; however, no significant differences between subsequent days were noted (Figures 3F,H). Pchlide low-temperature emission spectra obtained from 3- and 4-day etiolated seedlings showed an additional red-shifted band with a maximum at around 670 nm (Figure 3I). This peak was previously attributed to the aggregates of free Pchlide (Myśliwa-Kurdziel et al., 2013). Therefore, in this case, the proportion of 632/652 nm bands did not properly reflect the ratio of free and complex-bound Pchlide. In the case of seedlings etiolated for 5 and 6 days, the Pchlide spectra were similar, with a small decrease in the signal coming from the Pchlide:LPOR:NADPH in the latter (Figure 3I). In experiments on the Arabidopsis mutants, we decided to use samples collected during the 5th day of etiolation showing a large PLB and a typical Pchlide fluorescence pattern with a relatively high contribution of the photoactive Pchlide.

### 2D PLB Morphology in Plants With Disturbed Levels of Pigments and Polyprenols

**FIGURE 4:**
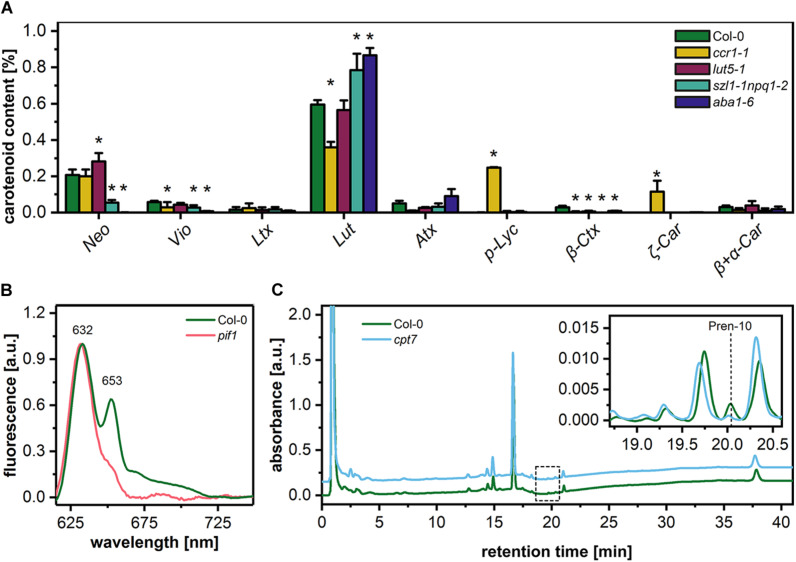
Percentage contribution of particular carotenoids (Neo – neoxanthin, Vio – violaxanthin, Ltx – luteoxanthin, Lut – lutein, Atx – anteraxanthin, p-Lyc – pro-lycopene, Ctx – cryptoxanthin, Car – carotene) in the total carotenoid pool of 5-day etiolated seedlings of Col-0 and carotenoid deficient Arabidopsis mutants (*ccr1-1*, *lut5-1*, *szl1-1npq1-2*, *aba1-6*) **(A)**; data are mean values ± SD from at least three independent experiments; results marked with asterisk on the top of the bar differ significantly at *p* = 0.05 from Col-0. Representative low-temperature (77 K) fluorescence emission spectra (excitation at 440 nm) of cotyledons and the upper 2 mm of hypocotyl of 5-day etiolated Col-0 and *pif1* seedlings **(B)**; all spectra were normalized to the maximal value. HPLC/UV chromatograms of total polyprenol extracts obtained from 5-day etiolated seedlings of Col-0 and *cpt7* mutant **(C)**; the main polyprenol (Pren-10) peak is indicated on the representative chromatograms.

To elucidate the structural role of pigment and polyprenol components in the formation of PLB structure, we selected different Arabidopsis mutants with disturbed levels of particular PLB membrane components (Figure 4). The first group of mutants was aberrant in the carotenoid composition. The *ccr1-1* mutant had a significantly decreased lutein (Lut) and violaxanthin (Vio) contribution in the total carotenoid pool. Moreover, the accumulation of pro-lycopene (p-Lyc), as well as ζ carotene (ζ-Car), was registered in the *ccr1-1* mutant only (Figure 4A). In the carotenoid composition of the *lut5-1* seedlings, the most significant difference compared with the Col-0 ecotype was an increase in the neoxanthin (Neo) contribution to the total carotenoid pool (Figure 4A). The opposite effect was observed in the *szl1-1npq1-2* plants in which a decrease in the Neo contribution was accompanied by lowered levels of Vio and a substantial increase in the Lut content compared with Col-0 plants (Figure 4A). We also registered Lut-overaccumulation in *aba1-6* plants in which the complete depletion of Neo and Vio was detected (Figure 4A). Moreover, a small amount of β-cryptoxanthin (β-Ctx) was detected in Col-0 plants, and only traces of this pigment were registered in the examined mutants. Pchlide is the most abundant precursor pigment of PLBs, and, as we mentioned above, plants depleted of this molecule do not form the PLB paracrystalline lattice. In this study, we used the Pchlide over-accumulating *pif1* mutant (Huq et al., 2004), which accumulates the non-photoconvertible form of this precursor pigment (Figure 4B). This enables us to reveal if increased Pchlide level can modulate the PLB morphology. To decipher the role of polyprenols in the PLB formation, we used the *cpt7* mutant deficient in plastidial polyprenols (Figure 4C).

**FIGURE 5:**
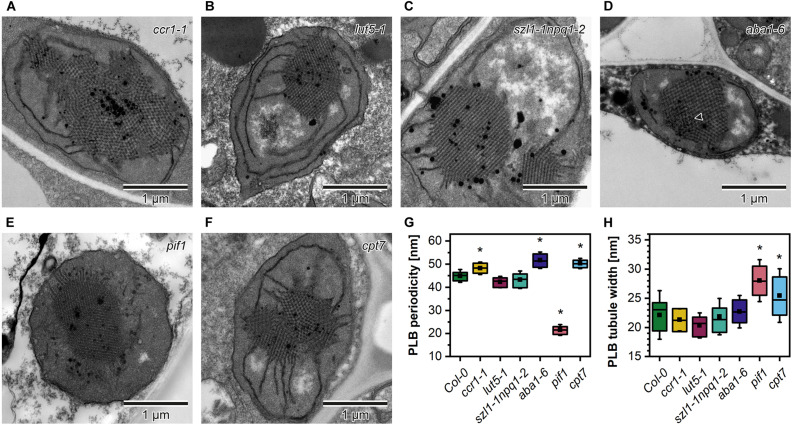
Ultrastructure of cotyledon etioplasts of 5-day etiolated seedlings of Arabidopsis carotenoid deficient mutants (*ccr1-1*, *lut5-1*, *szl1-1npq1-2*, *aba1-6*) **(A–F)**; black arrowhead – distortion in the bicontinuous structure of the *aba1-6* prolamellar body (PLB). Measurements of the PLB structural parameters **(G,H)**; description of the chart box as in Figure 2; results marked with asterisk on the top of the boxplot differ significantly at *p* = 0.05 from Col-0.

**FIGURE 6:**
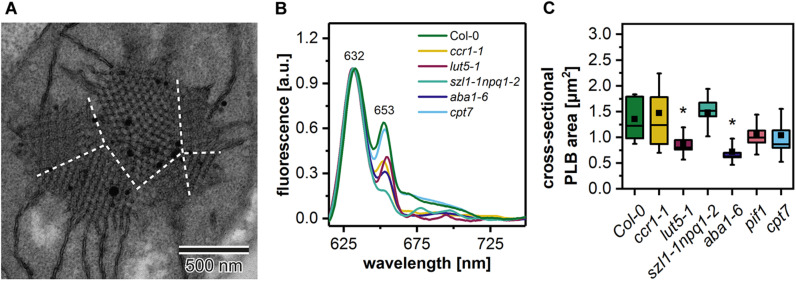
Electron micrograph of a “polycrystalline” prolamellar body (PLB) **(A)**; dashed white lines separate PLB regions arranged at different angles; note that PLB image is an enlargement of the Figure 5F micrograph. Representative low-temperature (77 K) fluorescence emission spectra (excitation at 440 nm) of cotyledons and the upper 2 mm of hypocotyl of 5-day etiolated Col-0 and *ccr1-1*, *lut5-1*, *szl1-1npq1-2*, *aba1-6, cpt7* seedlings **(B)**; all spectra were normalized to the maximal value. Measurements of the PLB cross-sectional area **(C)**; description of the chart box as in Figure 2; results marked with asterisk on the top of the boxplot differ significantly at *p* = 0.05 from Col-0.

In the ultrastructure of all examined mutant etioplasts, we observed well-differentiated PLB arrangements (Figure 5). Qualitative analysis revealed that in *aba1-6* plants, local disturbances in the paracrystalline structure of the PLB network were present (Figure 5D). In other mutants, we observed a uniform bicontinuous PLB structure. However, in all examined genotypes, particular PLBs were composed of several identical periodic configurations connected at different angles forming pseudo polycrystalline arrangements (Figures 5A–F, 6A). In the quantitative analysis, we established that the PLB periodicity is most severely affected in the *pif1* plants in which high PLB compactness was observed (Figures 5E,G). Moreover, due to a substantial increase in the diameter of the PLB-building tubule (Figure 5H and Supplementary Figures 1E,F), the observed PLB configuration in the *pif1* mutant was highly balanced in terms of outer and inner channel widths (Figures 5G,H, 8G,H). Such an arrangement is typical for many nature-occurring cubic membrane structures (reviewed in Almsherqi et al., 2009); however, it has not been observed yet in the etioplast PLBs. Slightly increased PLB periodicity was detected in the *ccr1-1, aba1-6*, and *cpt7* mutants compared with Col-0 plants (Figure 5G). In the case of *ccr1-1 and cpt7* plants, a decreased PLB compactness was accompanied by an increase in the PLB tubule width (Figure 5H). We did not recognize any correlation between the PLB organization, its cross-sectional size, and the Pchlide fluorescence pattern in the analyzed plants (Figures 5A–F, 6B,C).

### The Spatial Arrangement of PLB in Plants With Pigment and Polyprenol Deficits

**FIGURE 7:**
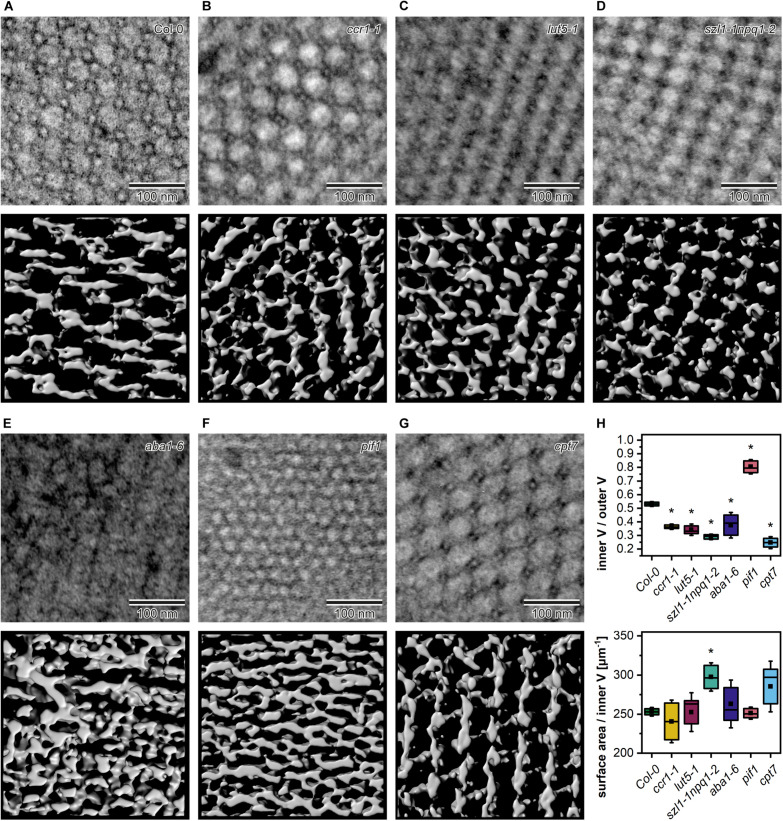
Middle layer of the tomography stack (upper panel) and rendered 3D membrane isosurface from the same region of the prolamellar body (PLB) (lower panel) of 5-day etiolated Col-0 and mutant (*ccr1-1*, *lut5-1*, *szl1-1npq1-2*, *aba1-6, pif1*, *cpt7*) seedlings **(A–G)**. Measurements of the spatial PLB parameters **(H)**; V – volume; description of the chart box as in Figure 2; results marked with asterisk on the top of the boxplot differ significantly at *p* = 0.05 from Col-0.

We studied the PLB organization in 3D using the ET method (Figure 7). Based on the reconstructed tomography stacks, we rendered isosurfaces reflecting the PLB lattice configuration visible from the angle determined by the cross-section orientation (Figures 7A–G). Due to the randomness of TEM/ET sample-cutting, the PLB lattices of different genotypes were analyzed from various angles (Figures 7A–G). Weakness of this method had, however, a limited impact on the calculated spatial PLB parameters (Figure 7H). It was due to the fact that every tomogram (at least 3) obtained for a particular genotype was positioned at different angles, and the visualized region typically covered polycrystalline configurations. The inner/outer volume ratio (*I*_v_/*O*_v_) (Supplementary Figures 1G–I) is a spatial parameter reflecting the balance between inner and outer PLB regions that represent the lumen and stroma compartment, respectively. The *I*_v_/*O*_v_ ratio calculated from the 3D models was significantly increased in the *pif1* plants, reaching the value of about 0.85, which indicated an increased balance between the PLB water channels of this mutant (Figure 7H). On the contrary, the *I*_v_/*O*_v_ ratio was decreased in all other examined mutants, *cpt7* in particular. The local PLB structural aberrations resulted in a higher standard deviation (SD) of the *I*_v_/*O*_v_ parameter in *aba1-6* plants compared to other analyzed genotypes. In terms of the surface area to *I*_v_ ratio, significantly different values from Col-0 were registered in the *szl1-1npq1-2* plants only (Figure 7H).

### The Relevance of Theoretical 3D Models in the Analysis of Spatial PLB Parameters

**FIGURE 8:**
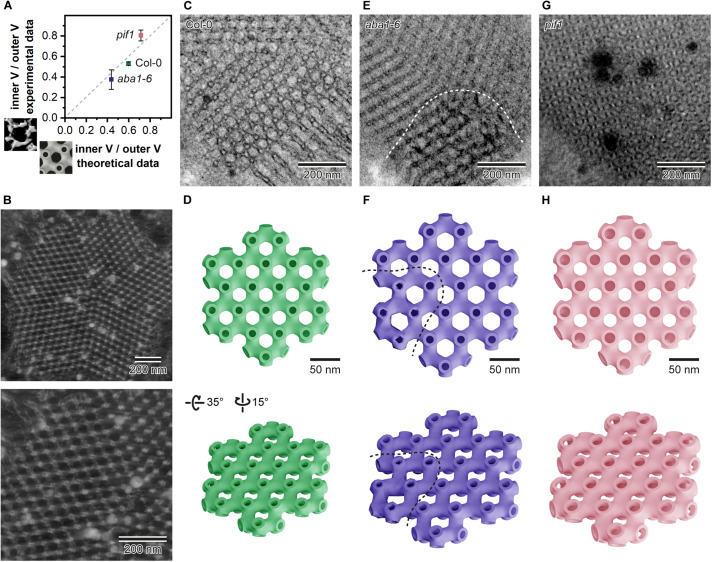
Comparison of the inner/outer volume (V) ratio obtained from the isosurface models based on tomography data (Y axis) and data generated from the theoretical 3D models rendered according to the prolamellar body (PLB) lattice dimensions calculated from the 90 nm thick 2D TEM sections for Col-0, *aba1-6*, and *pif1* cotyledons **(A)**. Reconstructed exemplary PLB tomography volume displayed in Imaris software **(B)**; whole PLB (upper panel) and magnified lattice fragment (lower panel). Exemplary micrograph of the Col-0 PLB cut at an angle showing the hexagonal pattern of the lattice **(C)**. Theoretical model of a single layer of the Col-0 PLB network seen from two different angles (upper/lower panel) **(D)**. Exemplary micrograph of the *aba1-6* PLB showing regular bicontinuous configuration and a region exhibiting distortions in the lattice structure separated by a dashed white curved line **(E)**; note that the orientation of the *aba1-6* PLB cut is not parallel to the one in Col-0 shown in panel C. Theoretical model of a single layer of the *aba1-6* PLB network seen from two different angles (upper/lower panel); regular and aberrant region of the model is divided by a dashed curved line **(F)**. Exemplary micrograph of the *pif1* PLB cut at an angle showing a hexagonal pattern of the balanced lattice of this mutant **(G)**. Theoretical model of a single layer of the *pif1* PLB network seen from two different angles (upper/lower panel) **(H)**. Note that all theoretical models were rendered using mean values calculated form the multiple 2D PLB cross-sections of respective genotypes; membrane thickness in all theoretical models was fixed to 6 nm.

Based on measurements of the PLB structural parameters from the TEM cross-sections, we generated 3D theoretical models of the PLB lattice in Col-0 plants and two mutants showing the most significant aberrations in the PLB nano-morphology (*aba1-6* and *pif1*) (Figure 8). Similarly to data obtained from the tomography-based 3D isosurfaces, we calculated the *I*_v_/*O*_v_ ratio for the theoretical models (Figure 8A). The results based on the experimental and theoretical models correlate quite accurately (less than 10% differences) (Figure 8A). This indicates that, in terms of spatial parameters, theoretical 3D models of highly repetitive cubic structures based on 2D TEM measurements can serve as a reasonable replacement for time and money consuming tomography experiments (Figures 8B–H). Such assumption was confirmed for significantly different PLB structures of three selected genotypes.

## Discussion

This work concerns the interplay between the structure and composition of the cubic membranes, as exemplified by the influence of carotenoid and polyprenol on the PLB nano-morphology. Therefore, in a broader context, our studies provide a better understanding of membrane folding at the molecular level. This remains one of the key topics in cell biology, still poorly understood.

### The Importance of Experimental Setup in PLB Morphology Studies

Although the skotomorphogenesis (etiolated growth) has been a subject of many studies and the use of the etioplast model to track the chloroplast biogenesis at different levels of their organization is quite common (reviewed in Adam et al., 2011; Gabruk and Mysliwa-Kurdziel, 2015; Pogson et al., 2015; Rast et al., 2015; Mechela et al., 2019; Armarego-Marriott et al., 2020), no uniform model of plant cultivation was established. In this study, we examined whether different, typically used, conditions of Arabidopsis etiolation could impact the PLB development, and therefore, be the source of literature discrepancies.

In many studies on the Arabidopsis etiolated seedlings, the Suc-supplement nutrition media is applied. Probably, it is mostly related to the fact that the addition of Suc results in an increase of the seedling size by promoting cell proliferation and in a delay of the switch to cell expansion via the glucose-6-phosphate transporter pathway (Van Dingenen et al., 2016). Due to very small size of the Arabidopsis etiolated seedlings such procedure is very useful in, e.g., biochemical experiments that require sufficiently large sample biomass. However, it should be noticed that Suc plays also a signaling role in plastid development, affecting the chloroplast structure and biogenesis in the early stages of leaf development (Van Dingenen et al., 2016). Similarly, using etiolated seedlings, we showed that the PLB nano-morphology and etioplast spectral properties are altered in plants growing on Suc-supplemented media (Figure 2). The observed changes in the chloroplast development are probably mainly related to the repression of the plastid and nucleus-encoded photosynthetic proteins (Krapp et al., 1993). The influence of Suc on the regulation of the biosynthesis of components localized in PLB remains to be tested. However, based on the different structural responses of etioplasts on various nutrition media (Figure 2), we argue for using experimental setup in which no additional carbon source is applied. Only such approach will enable reliable use of dark-grown seedlings in further de-etiolation experiments.

The structural pathways of PLB formation during the process of skotomorphogenesis have been a subject of several studies performed on bean, pea, oat, and tobacco plants (Von Wettstein, 1967; Ikeda, 1968; Weier and Brown, 1970; Klein and Schiff, 1972; Kesselmeier, 1980; Lütz, 1981; Mostowska, 1986). So far, however, no consensus on the way of the formation of the PLB cubic structure has been reached. The obtained data suggest different possible formation patterns, including fusion of vesicles or contraction of porous membranes into regularly ordered tubules. Studies on PLB formation are particularly difficult because PLB structures are found even in the early stages of etiolation (Kowalewska and Mostowska, 2016). Similarly, in this study, we showed that the developed bicontinuous regular PLB structure is detected already after 3 days of etiolation (Figure 3), arguing for a short timescale of the PLB formation. Such observations suggest a rapid PLB accumulation, which is performed via an organized phase transition rather than a unit-by-unit way of assembly proposed earlier as a possible general way of cubic membrane folding (Landh, 1996). However, further, highly resolved studies are required to address this topic. In subsequent days of etiolation the PLB enlarges, the network periodicity undergoes a gradual decrease, and the Pchlide fluorescence pattern changes (Figure 3). Therefore, particular attention should be drawn to the comparison of the structural and spectral results obtained for different dark-growth times. The same caution should be applied for seedling region subjected for further analysis (Figure 1) to exclude non-uniform plastid pool.

### The Role of Pigments and Polyprenols in the PLB Structure Assembly

Carotenoids and chlorophylls are essential components of the thylakoid network of chloroplasts, and their complete depletion is lethal. In the thylakoid membranes, these pigments are mainly bound with the photosynthetic proteins and they together play both structural and functional roles in the establishment of the photosynthetic efficiency (Lundqvist and Franckowiak, 2003; Dall’Osto et al., 2014). However, it has been estimated that about 15% of the chloroplast thylakoid carotenoids in Arabidopsis are localized in the lipid matrix phase influencing the physical properties of the membrane (Dall’Osto et al., 2010). Since PLB membranes are deficient in such pigment-binding proteins (Blomqvist et al., 2008), probably almost all carotenoids of etioplasts are located directly in the lipid phase of PLBs and prothylakoids. Therefore, their direct influence on the PLB structure might be even more pronounced than in the lamellar arrangement of the chloroplast thylakoids. It should be stressed that at least part of the PLB carotenoid pool could be located in the plastoglobules directly interconnected with the cubic lattice. The presence of particular carotenoids was confirmed in plastoglobules of chromo- and chloroplasts; however, the pigment composition of etioplast plastoglobules has not yet been identified (Ytterberg et al., 2006; Van Wijk and Kessler, 2017).

We tested four Arabidopsis mutants (*ccr1-1*, *lut5-1*, *szl1-1npq1-2*, *aba1-6*) deficient in different carotenoid classes (Figure 4). These mutants were earlier studied using fully developed plants, mainly in the context of the efficiency of the photosynthetic apparatus (e.g., Niyogi et al., 1998; Park et al., 2002; Kim and DellaPenna, 2006; Li et al., 2009). However, their role in the formation of the chloroplast thylakoid network structure is still elusive. Regarding the PLB organization, we registered the most pronounced changes in *aba1-6* plants, which over-accumulated Lut and were depleted in Neo and Vio (Figure 4). Local disturbances in bicontinuous PLB configurations were visible in this mutant (Figures 5, 7, 8). An increase in Lut contribution was earlier attributed to a decrease in membrane fluidity (Gruszecki and Strzałka, 2005), due mostly to specific Lut structure enabling its molecules in the all-*trans* conformation to adopt two orthogonal orientations in the lipid bilayer (Sujak et al., 1999). The role of Lut- and DGDG-dependent membrane rigidification of dark-chilled cucumber etioplasts in the switch from “close” to “open” PLB structure was shown before; however, no disturbances in the bicontinuous PLB lattice configuration were registered (Skupień et al., 2017). Such results, together with the fact that no aberration in the PLB structure was detected in the *szl1-1npq1-2* plants (Figures 5, 7), points to limited role of Lut over-accumulation in the maintenance of the PLB cubic configuration. We rather presume that the β-β-xanthophylls (Neo, Vio), which are depleted in *aba1-6* seedlings, could play a stabilizing role in the PLB formation. However, it is worth noting that in the *aba1-6* plants, characterized by a decreased level of the abscisic acid (Peskan-Berghöfer et al., 2015), levels of other PLB membrane components might be disturbed due to a broad regulatory role of this phytohormone (Wasilewska et al., 2008). Therefore, further studies are required to address this topic both in the context of etioplast PLBs but also of the chloroplast thylakoid membranes. Surprisingly, although the carotenoid composition is severely affected in the *ccr1-1* plants (Figure 4), the PLB in this mutant is fully developed and regularly arranged (Figures 5, 7). It was previously established that in the *ccr2* plants, the formation of PLB is completely arrested (Park et al., 2002; Cazzonelli et al., 2020). Such fact was attributed to the accumulation of *cis*-carotenes – p-Lyc and pro-neurosporene in particular. Park et al. (2002) presented a theoretical scheme showing how the steeped structure of *cis*-carotenes could prevent formation of the membrane curvature necessary for the PLB development. Restoration of the PLB structure is possible in the *ccr2* mutant via further mutation in the ζ-carotene isomerase (ZISO), resulting in the block of *cis*-carotenes synthesis (Cazzonelli et al., 2020). The reason for the wild-type like PLB formation in the *cis*-carotenoid accumulating *ccr1-1* mutant remains unclear (Figures 4, 5). We hypothesize however, that milder *ccr1-1* phenotype compared to *ccr2* plants, visible as only a partial decrease of the amount of Lut and a lower abundance of *cis*-carotenoids (pro-neurosporene and neurosporene particularly), is the most probable reason for PLB survival in the *ccr1-1* plants. Moreover, it is possible that accumulated *cis*-carotenoids do not form any type of steric hindrance for the PLB membrane bending. We can speculate that lack of PLB in the *ccr2* plants is related to other, not identified yet membrane component and its level regulated via an apo-carotenoid signaling pathway.

Pchlide is the most abundant in the diverse group of PLB pigments; its importance in the PLB formation was studied previously using plants with decreased levels of this precursor pigment. Here we used the Pchlide over-accumulating *pif1* plants to check whether increased Pchlide level can influence PLB structure. It was suggested before, that the complex-bound Pchlide is located in the PLB lattice, while free non-photoconvertible Pchlide is rather located in the prothylakoids, loosely arranged around PLB lattice (Lindsten et al., 1988). It was also proposed that an increased contribution of the photoconvertible Pchlide in the fluorescence spectra could serve as an adaptation mechanism maximizing the use of light during the Pchlide transformation and promoting efficient conversion of PLB to thylakoids (Aronsson et al., 2008). However, our studies on pigment and polyprenol deficient mutants did not reveal any pattern linking the PLB periodicity, its cross-sectional size, and the Pchlide 632/653 nm ratio (Figures 5, 6). This suggests that photo and non-photoconvertible Pchlide forms could be located both in PLB and prothylakoids. We showed that even in hypocotyl regions deprived of PLB structures, the fluorescence spectra have similar ratio of 632/653 nm peaks to the seedling shoot including whole hypocotyl and PLB-rich cotyledons (Figure 1). Therefore, Pchlide:LPOR:NADPH complex might be located in prothylakoids, which was also confirmed earlier on isolated and fractionated internal etioplast membranes of wheat (Ryberg and Sundqvist, 1982). Based on our structural and spectral analyses, we presume that free Pchlide overaccumulated in the *pif1* plants is located directly in the PLB lattice, causing an increase in its compactness. Similar spectral pattern together with comparable PLB periodicity value were detected earlier in *cop1/*PBO-1 line characterized by *cop1* mutation (lack of PLB structure and complex-bound Pchlide) and simultaneous overexpression of PORB protein rescuing PLB cubic arrangement and partial formation of Pchlide:LPOR:NADPH complex (Sperling et al., 1998). Probably Pchlide-dependent decrease in PLB periodicity is not directly related to the Pchlide molecule shape but is rather connected with the imbalance between different groups of the PLB building blocks, a relative decrease in the polar-lipid contribution particularly. Studies on ER-originating cubic membranes in the fibroblast cell culture lines revealed that over-accumulation of particular ER-resident proteins markedly changing the protein/lipid ratio results in formation of the cubic arrangements (Snapp et al., 2003; Almsherqi et al., 2006). Such observations of even distant biological species are particularly relevant for a better understanding of cubic membrane differentiation. This is based on the assumption that the formation and stability of membrane configurations can be understood from the same basic principles because formation mechanisms are probably dictated by the charge and geometry of molecules rather than a specific amino acid sequence or lipid class.

Finally, we tested the role of plastid-located polyprenols in the PLB formation. The possible structural role of polyprenols in the chloroplast thylakoid membranes was raised before (Akhtar et al., 2017); however, no data concerning their potential role in the PLB formation were available. Due to a lower protein/lipid ratio in the etioplast membranes compared to thylakoids (Solymosi and Aronsson, 2013), it is highly probable that polar and non-polar lipid components play crucial role in the establishment of the cubic arrangement. However, diminish in plastidic polyprenols in the *cpt7* mutant had a limited influence on the PLB arrangement causing only a slight decrease in the PLB compactness (Figures 4, 5). It might be related to the fact that even in etiolated seedlings of wt plants, polyprenol level is relatively low, and the abundance of only main plastidial polyprenol (Pren-10) was registered. Such result is consistent with earlier studies indicating induction of polyprenol accumulation on light and substantial increase of their content in photosynthetic tissue during leaf senescence (reviewed in Swiezewska and Danikiewicz, 2005).

Studies on the 3D PLB nano-morphology are particularly complicated due to small dimensions of the PLB lattice. Previously, a template matching technique was applied to predict the spatial arrangement of cubic membranes visible in the TEM cross-sections (Deng and Mieczkowski, 1998). However, no spatial parameters of the predicted structures nor a confirmation in the 3D experimental data were provided. Here, we obtained the actual 3D models of the PLB lattices from the ET experiments and compared the structural parameters with the ones generated from rendered spatial theoretical models based on the 2D TEM information (Figure 8). High level of correlation of these results gives an experimental evidence directly justifying further development of the matching technique. Progress in this method will enable prediction of the cubic spatial parameters in a faster and more efficient way than by using time and money-consuming 3D electron microscopy experiments.

## Conclusion

This study provides a comprehensive analysis of the influence of different experimental setups typically used in etiolation/de-etiolation studies on the PLB nano-morphology. Our results point to the important role of Suc supplementation of the nutrition media and the duration of etiolation on the compactness and size of the PLB, as well as etioplast spectral properties. Therefore, particular attention should be drawn whether data obtained in different etiolation conditions are reliably comparable. In our studies using different Arabidopsis mutants with disturbed levels of the PLB pigments and polyprenols, we pointed out the important role of β-β-xanthophylls in the stabilization of the bicontinuous PLB structure, as well as the Pchlide level in the control of PLB compactness. This study broadens our understanding of the mechanisms governing cubic membrane formation, shedding light on the role of other than polar lipid and protein components in the cubic structure development. Further studies should consider how the disturbed PLB structure can influence the process of the tubular-lamellar transformation taking place during the chloroplast biogenesis. Irregular, over-compacted, or very loosely arranged PLB structure could substantially retard the chloroplast biogenesis via, e.g., trapping of molecules in hyperbended membranes or providing a limited lipid reservoir for the developing structures.

## Data Availability Statement

The raw data supporting the conclusions of this article will be made available by the authors, without undue reservation.

## Author Contributions

ŁK and AM provided the conception of the manuscript. MB, ŁK, RM, DB, and JS performed experiments. MB, ŁK, RM, and DB analyzed and processed data. ŁK coordinated the project. ŁK wrote the manuscript with a contribution of AM and DB. AM secured funding. All authors contributed to the article and approved the submitted version.

## Conflict of Interest

The authors declare that the research was conducted in the absence of any commercial or financial relationships that could be construed as a potential conflict of interest.
