# Supplementary material for: Spatial Nano-Morphology of the Prolamellar Body in Etiolated Arabidopsis thaliana Plants With Disturbed Pigment and Polyprenol Composition
Source: Front Cell Dev Biol. 2020 Oct 8;8:586628. doi: 10.3389/fcell.2020.586628 (PMC7578251; doi:10.3389/fcell.2020.586628)
Supplement: Supplementary file 1 [file Data_Sheet_1.pdf]

SUPPLEMENTARY MATERIAL

Spatial nano-morphology of the prolamellar body in etiolated *Arabidopsis thaliana* plants with disturbed pigment and polyprenol composition

Michał Bykowski[1], Radosław Mazur[2], Daniel Buszewicz[3], Joanna Szach[1], Agnieszka Mostowska[1] and Łucja Kowalewska[1*]

[1]Department of Plant Anatomy and Cytology, Institute of Plant Experimental Biology and Biotechnology, Faculty of Biology, University of Warsaw, Warsaw, Poland

[2]Department of Metabolic Regulation, Institute of Biochemistry, Faculty of Biology, University of Warsaw, Warsaw, Poland

[3]Institute of Biochemistry and Biophysics, Polish Academy of Sciences, Warsaw, Poland

*** Correspondence:**
Corresponding Author
lucja.kowalewska@uw.edu.pl

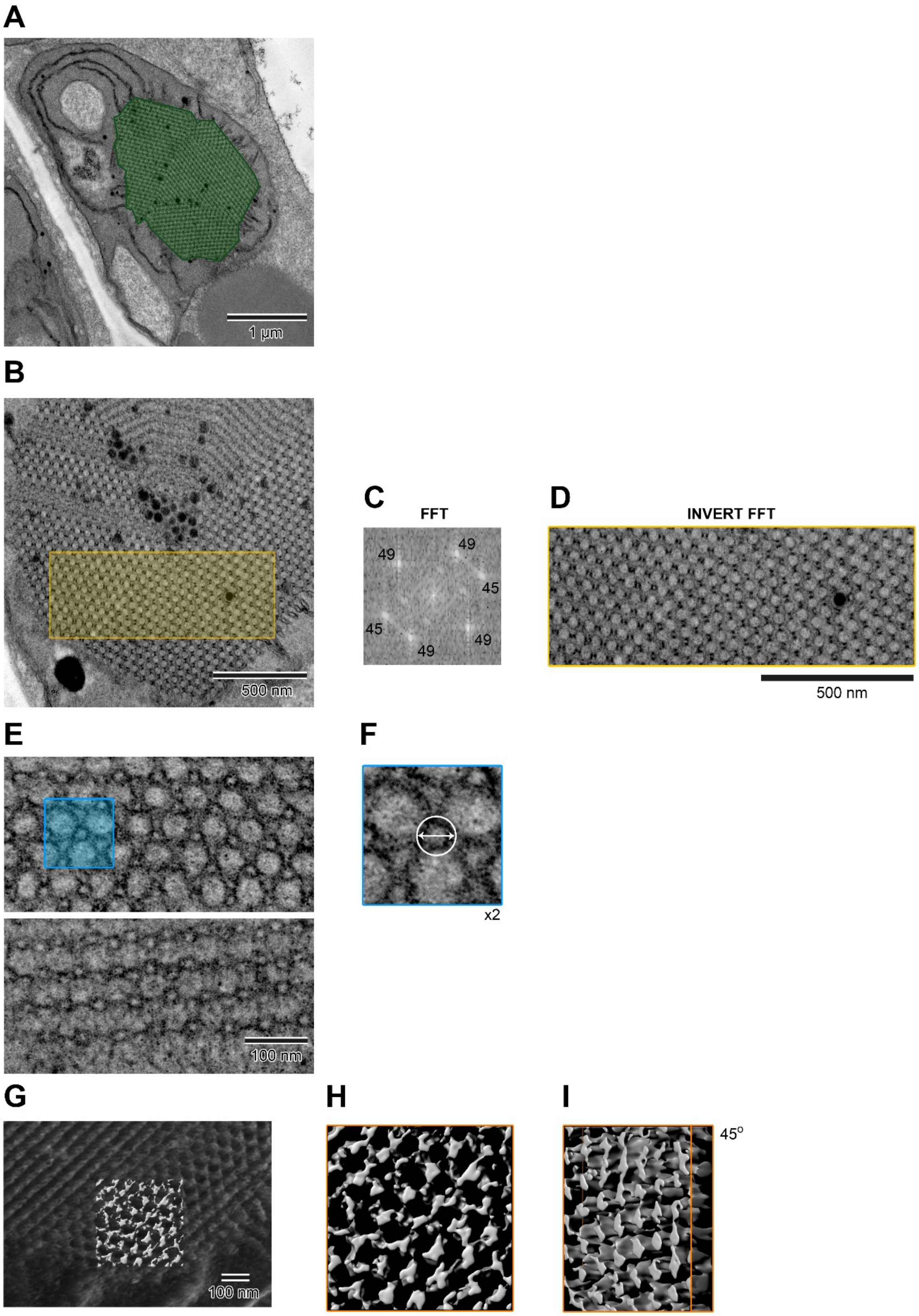

Supplementary Figure 1.

Micrograph of Arabidopsis etioplast with a polygon shape (green) showing tracing of prolamellar body (PLB) cross-sectional area (A). Micrograph of Arabidopsis PLB with a yellow rectangle (B) displaying exemplary region used for Fast Fourier Transform (FFT) (C, D), FFT

radius of points position represents the periodicity value in nm (C) and corresponds to the distances between the center of the neighboring cross-sectional hexagonal units. Images of Arabidopsis PLB showing lattice orientations (upper and lower panels) enabling tracing of the PLB tubule width (E), magnified region marked with blue square (E) showing details of diameter determination (F). Volume of the PLB rendered based on tomography data with a cuboidal region of interest showing PLB isosurface (G), magnified modeled isosurface visible from different angles (H, I), cuboids of unified sizes were used to calculate inner and outer volumes of the PLB – inner volume is the volume inside the modeled area (gray) and outer volume is calculated as a cuboid total volume - minus - volume of the modeled area. Presented micrographs are exemplary data from the experimental set of Arabidopsis wt and mutant samples selected to show the methodology used to determine the ultrastructural PLB features.
